# Supplementary material for: Angelica sinensis polysaccharide as potential protectants against recurrent spontaneous abortion: focus on autophagy regulation
Source: Front Med (Lausanne). 2025 Jan 15;12:1522503. doi: 10.3389/fmed.2025.1522503 (PMC11774876; doi:10.3389/fmed.2025.1522503)
Supplement: Supplementary material S1 — The main instruments used during the LC-MS process, along with their models/specifications and manufacturers. [file Supplementary_file_1.zip › Metabolomics sequencing data FC1.2/4.多元统计分析/Permutation/Permutation(Exposure_Ctrl).pdf]

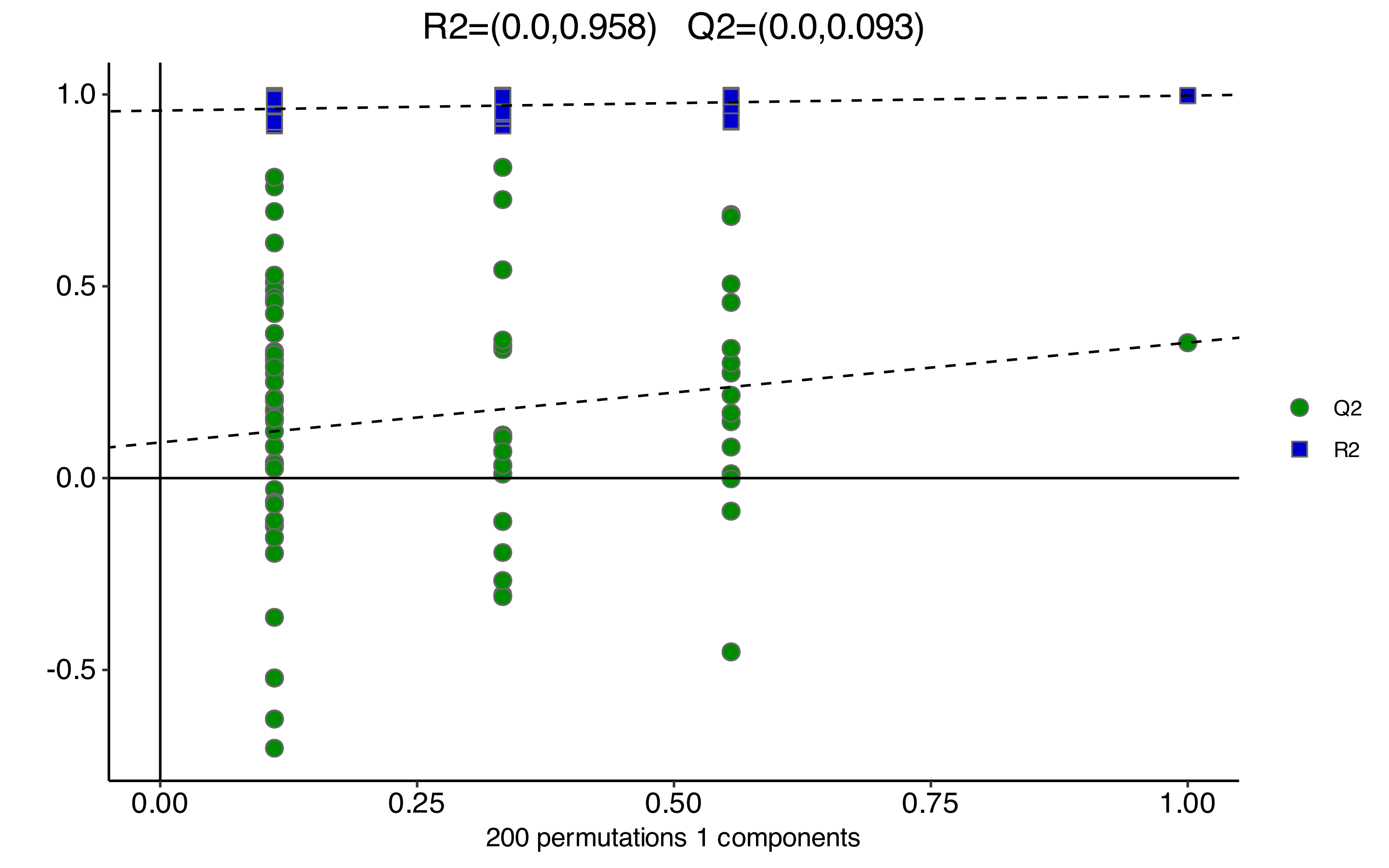

R2=(0.0,0.958) Q2=(0.0,0.093)
1.0
0.5
0.0
-0.5
0.00
0.25
0.50
0.75
1.00
200 permutations 1 components
Q2
R2
